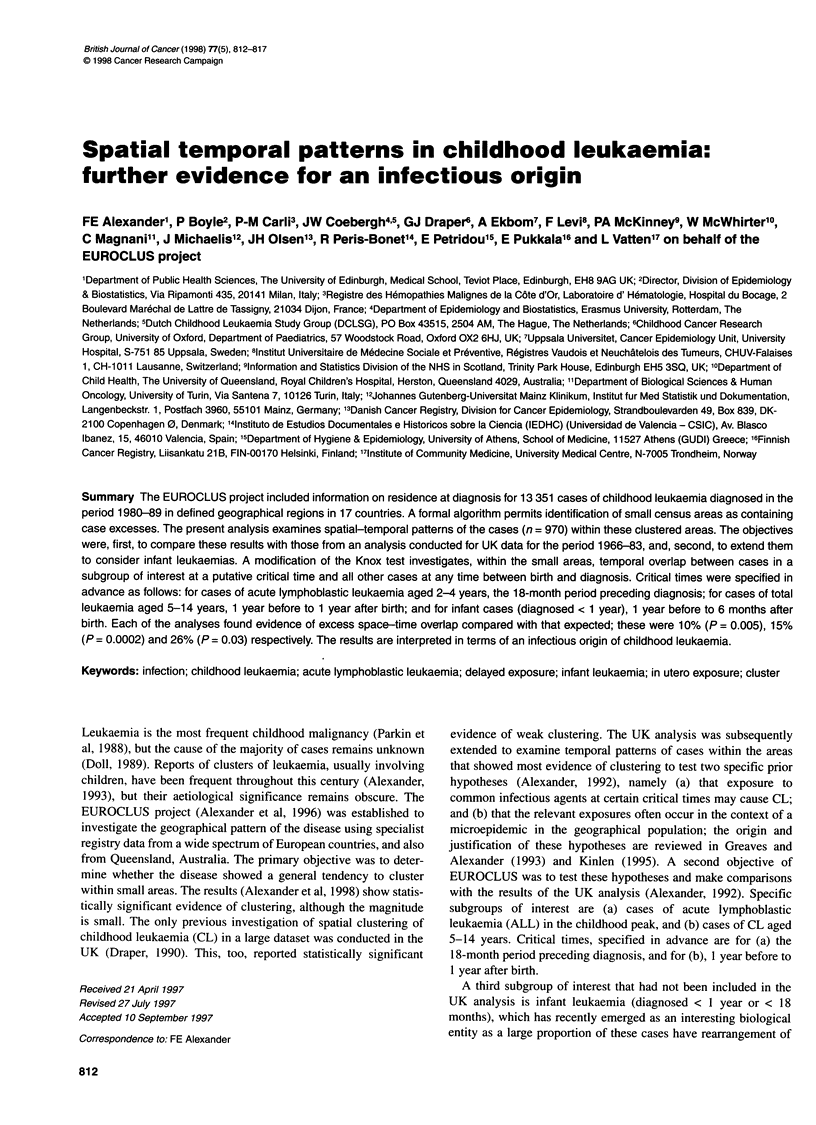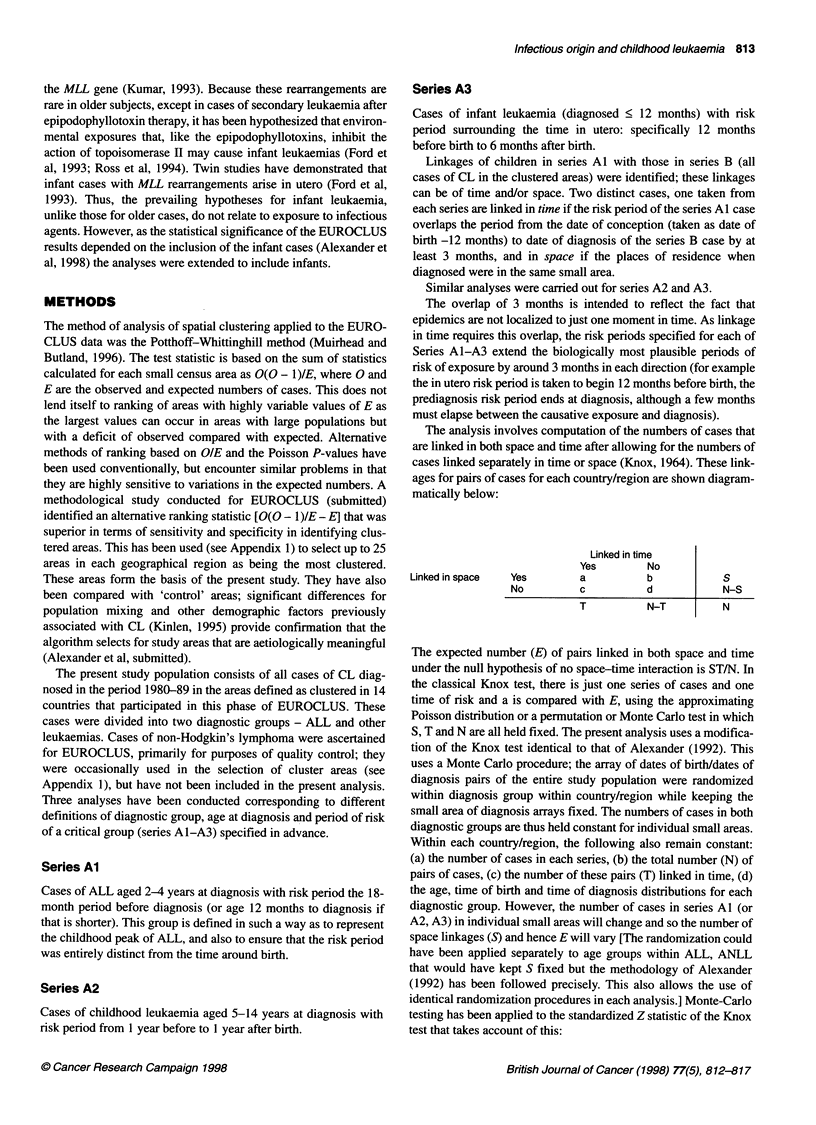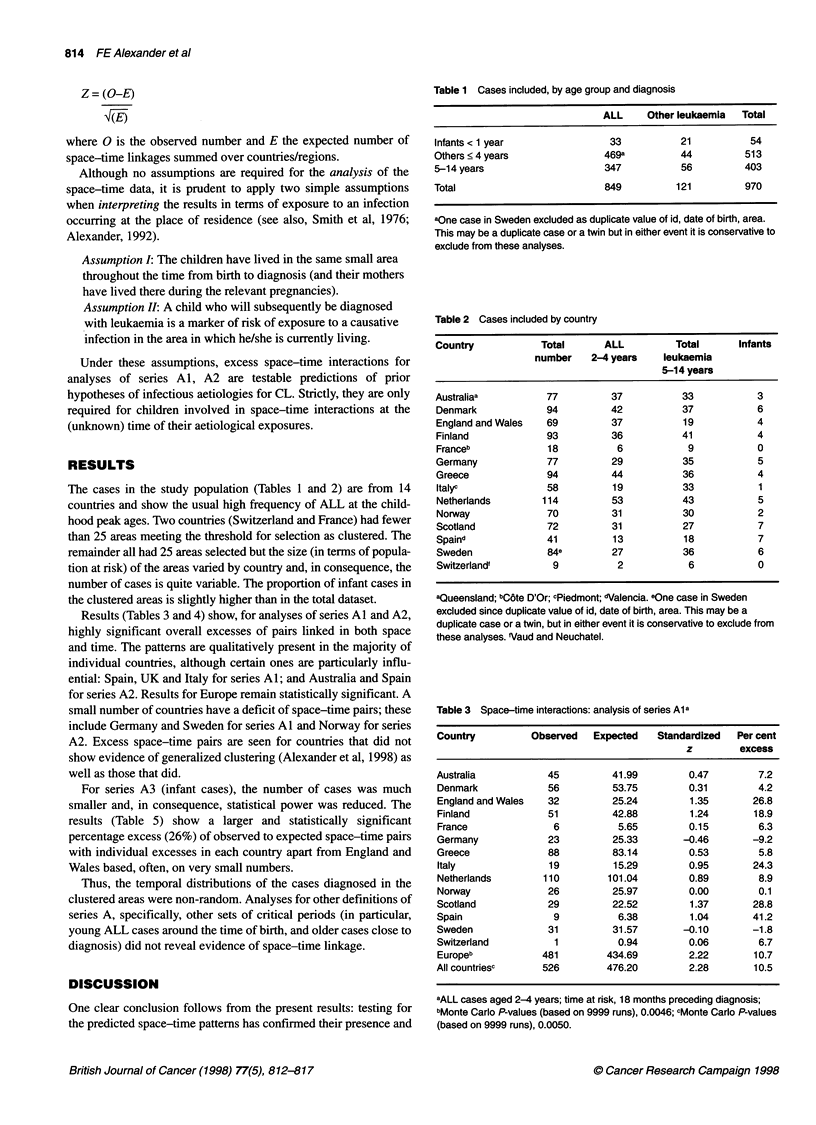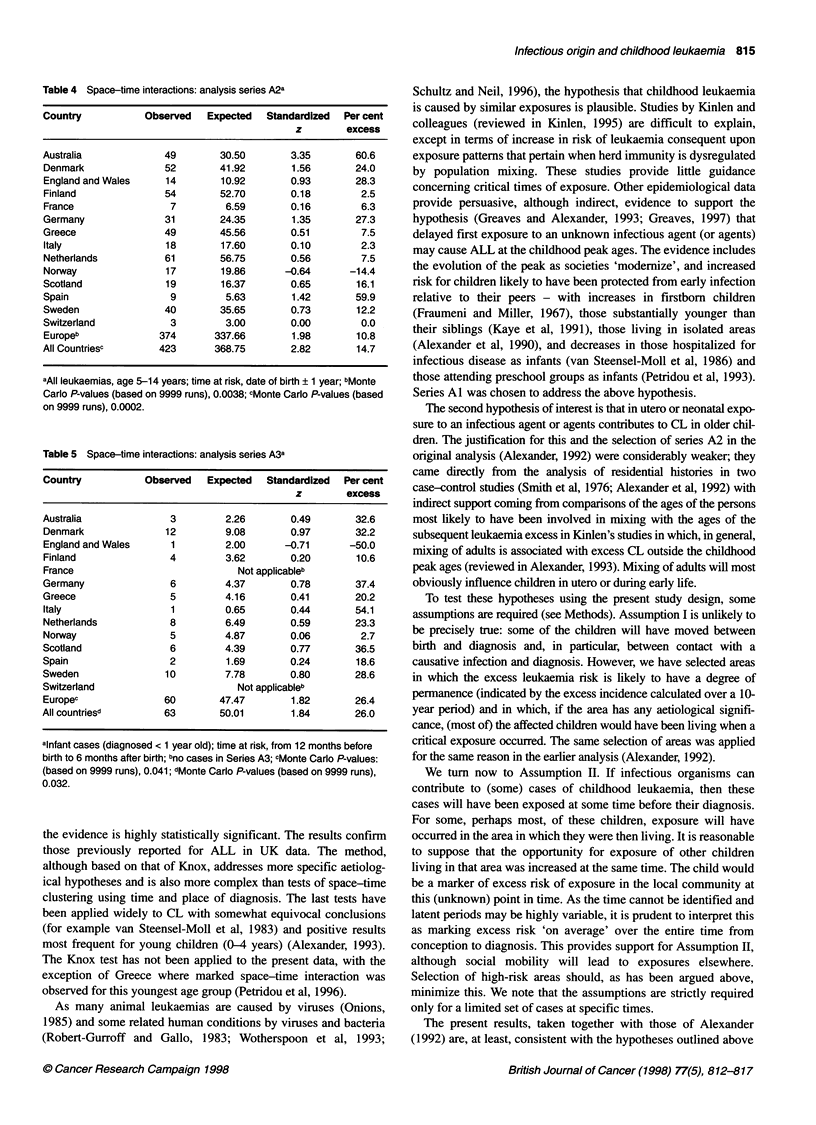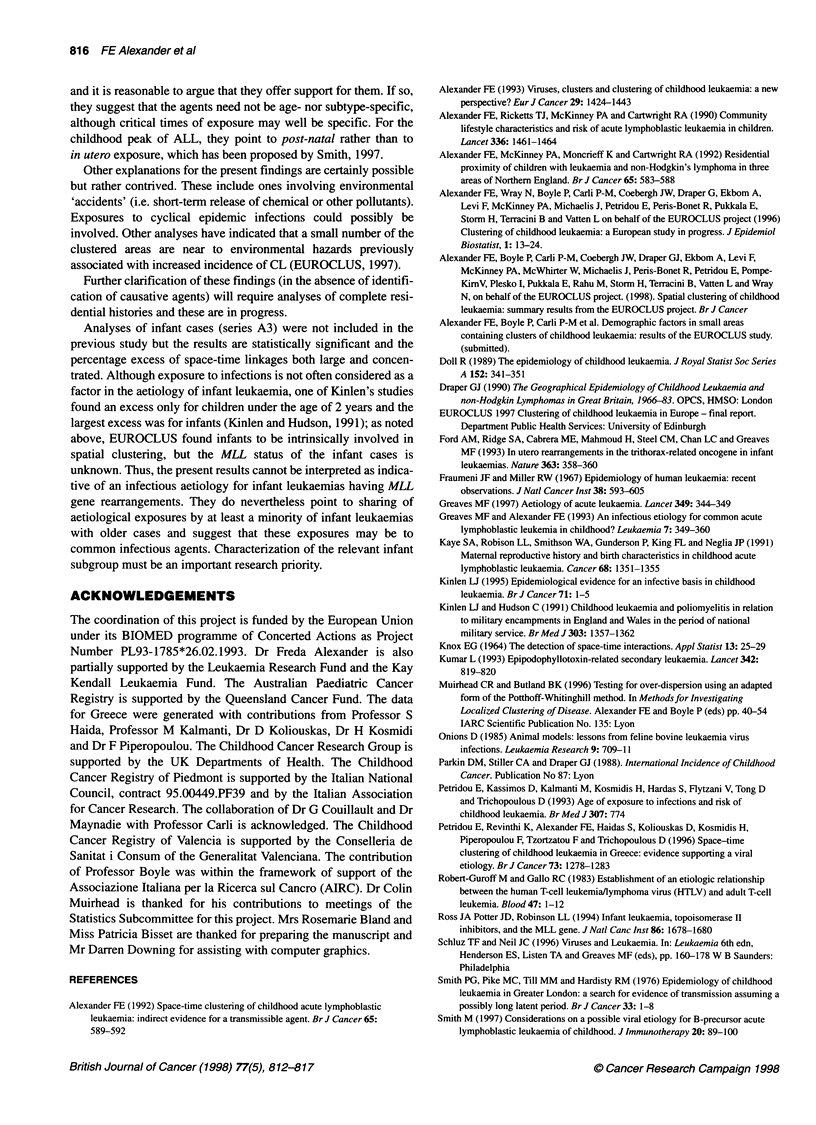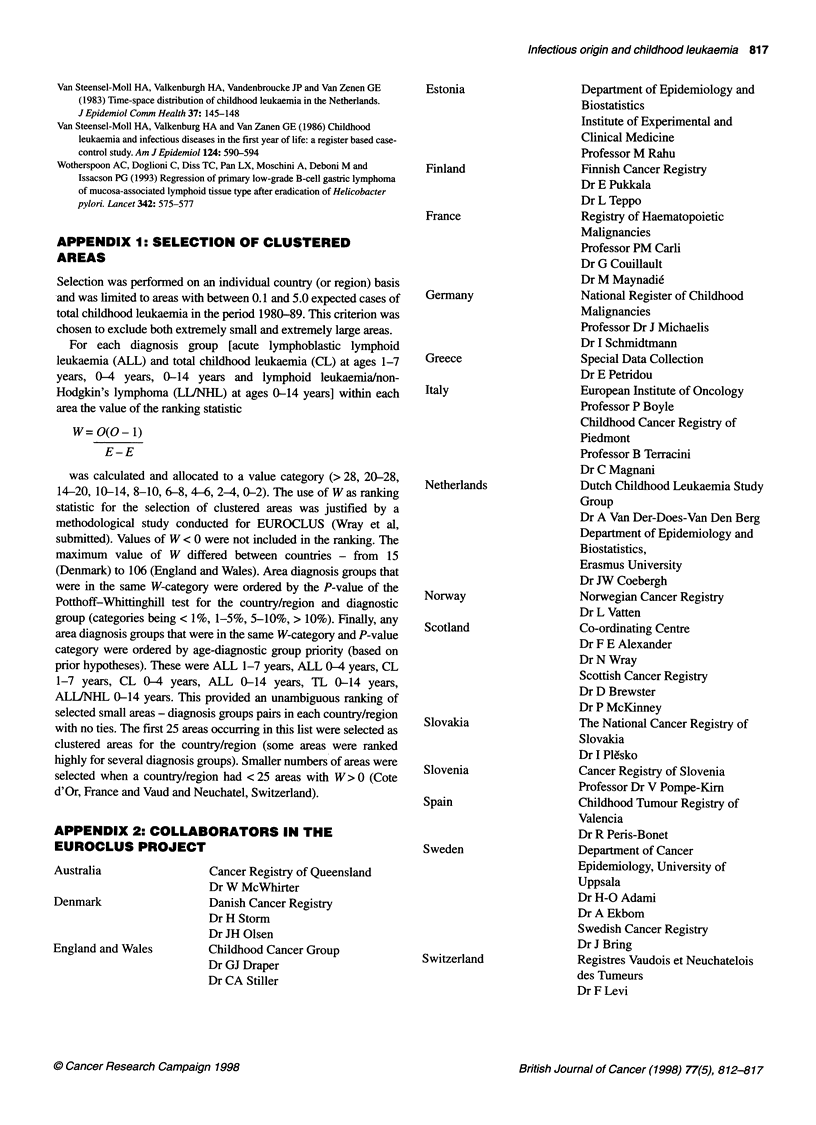# Spatial temporal patterns in childhood leukaemia: further evidence for an infectious origin. EUROCLUS project.

**DOI:** 10.1038/bjc.1998.132

**Published:** 1998-03

**Authors:** F. E. Alexander, P. Boyle, P. M. Carli, J. W. Coebergh, G. J. Draper, A. Ekbom, F. Levi, P. A. McKinney, W. McWhirter, C. Magnani, J. Michaelis, J. H. Olsen, R. Peris-Bonet, E. Petridou, E. Pukkala, L. Vatten

**Affiliations:** Department of Public Health Sciences, The University of Edinburgh, Medical School, UK.

## Abstract

The EUROCLUS project included information on residence at diagnosis for 13351 cases of childhood leukaemia diagnosed in the period 1980-89 in defined geographical regions in 17 countries. A formal algorithm permits identification of small census areas as containing case excesses. The present analysis examines spatial-temporal patterns of the cases (n = 970) within these clustered areas. The objectives were, first, to compare these results with those from an analysis conducted for UK data for the period 1966-83, and, second, to extend them to consider infant leukaemias. A modification of the Knox test investigates, within the small areas, temporal overlap between cases in a subgroup of interest at a putative critical time and all other cases at any time between birth and diagnosis. Critical times were specified in advance as follows: for cases of acute lymphoblastic leukaemia aged 2-4 years, the 18-month period preceding diagnosis; for cases of total leukaemia aged 5-14 years, 1 year before to 1 year after birth; and for infant cases (diagnosed < 1 year), 1 year before to 6 months after birth. Each of the analyses found evidence of excess space-time overlap compared with that expected; these were 10% (P = 0.005), 15% (P= 0.0002) and 26% (P= 0.03) respectively. The results are interpreted in terms of an infectious origin of childhood leukaemia.


*British Journal of Cancer* (1998) **77**(5), 812–817
© 1998 Cancer Research Campaign

### Spatial temporal patterns in childhood leukaemia: further evidence for an infectious origin

**FE Alexander[1], P Boyle[2], P-M Carli[3], JW Coebergh[4,5], GJ Draper[6], A Ekbom[7], F Levi[8], PA McKinney[9], W McWhirter[10], C Magnani[11], J Michaelis[12], JH Olsen[13], R Peris-Bonet[14], E Petridou[15], E Pukkala[16] and L Vatten[17] on behalf of the EUROCLUS project**

[1]Department of Public Health Sciences, The University of Edinburgh, Medical School, Teviot Place, Edinburgh, EH8 9AG UK; [2]Director, Division of Epidemiology & Biostatistics, Via Ripamonti 435, 20141 Milan, Italy; [3]Registre des Hémopathies Malignes de la Côte d'Or, Laboratoire d' Hématologie, Hospital du Bocage, 2 Boulevard Maréchal de Lattre de Tassigny, 21034 Dijon, France; [4]Department of Epidemiology and Biostatistics, Erasmus University, Rotterdam, The Netherlands; [5]Dutch Childhood Leukaemia Study Group (DCLSG), PO Box 43515, 2504 AM, The Hague, The Netherlands; [6]Childhood Cancer Research Group, University of Oxford, Department of Paediatrics, 57 Woodstock Road, Oxford OX2 6HJ, UK; [7]Uppsala Universitet, Cancer Epidemiology Unit, University Hospital, S-751 85 Uppsala, Sweden; [8]Institut Universitaire de Médecine Sociale et Préventive, Régistres Vaudois et Neuchâtelois des Tumeurs, CHUV-Falaises 1, CH-1011 Lausanne, Switzerland; [9]Information and Statistics Division of the NHS in Scotland, Trinity Park House, Edinburgh EH5 3SQ, UK; [10]Department of Child Health, The University of Queensland, Royal Children's Hospital, Herston, Queensland 4029, Australia; [11]Department of Biological Sciences & Human Oncology, University of Turin, Via Santena 7, 10126 Turin, Italy; [12]Johannes Gutenberg-Universitat Mainz Klinikum, Institut fur Med Statistik und Dokumentation, Langenbeckstr. 1, Postfach 3960, 55101 Mainz, Germany; [13]Danish Cancer Registry, Division for Cancer Epidemiology, Strandboulevarden 49, Box 839, DK-2100 Copenhagen Ø, Denmark; [14]Instituto de Estudios Documentales e Historicos sobre la Ciencia (IEDHC) (Universidad de Valencia – CSIC), Av. Blasco Ibanez, 15, 46010 Valencia, Spain; [15]Department of Hygiene & Epidemiology, University of Athens, School of Medicine, 11527 Athens (GUDI) Greece; [16]Finnish Cancer Registry, Liisankatu 21B, FIN-00170 Helsinki, Finland; [17]Institute of Community Medicine, University Medical Centre, N-7005 Trondheim, Norway

**Summary** The EUROCLUS project included information on residence at diagnosis for 13 351 cases of childhood leukaemia diagnosed in the period 1980–89 in defined geographical regions in 17 countries. A formal algorithm permits identification of small census areas as containing case excesses. The present analysis examines spatial–temporal patterns of the cases ($n = 970$) within these clustered areas. The objectives were, first, to compare these results with those from an analysis conducted for UK data for the period 1966–83, and, second, to extend them to consider infant leukaemias. A modification of the Knox test investigates, within the small areas, temporal overlap between cases in a subgroup of interest at a putative critical time and all other cases at any time between birth and diagnosis. Critical times were specified in advance as follows: for cases of acute lymphoblastic leukaemia aged 2–4 years, the 18-month period preceding diagnosis; for cases of total leukaemia aged 5–14 years, 1 year before to 1 year after birth; and for infant cases (diagnosed < 1 year), 1 year before to 6 months after birth. Each of the analyses found evidence of excess space–time overlap compared with that expected; these were 10% ($P = 0.005$), 15% ($P = 0.0002$) and 26% ($P = 0.03$) respectively. The results are interpreted in terms of an infectious origin of childhood leukaemia.

**Keywords:** infection; childhood leukaemia; acute lymphoblastic leukaemia; delayed exposure; infant leukaemia; in utero exposure; cluster

Leukaemia is the most frequent childhood malignancy (Parkin et al, 1988), but the cause of the majority of cases remains unknown (Doll, 1989). Reports of clusters of leukaemia, usually involving children, have been frequent throughout this century (Alexander, 1993), but their aetiological significance remains obscure. The EUROCLUS project (Alexander et al, 1996) was established to investigate the geographical pattern of the disease using specialist registry data from a wide spectrum of European countries, and also from Queensland, Australia. The primary objective was to determine whether the disease showed a general tendency to cluster within small areas. The results (Alexander et al, 1998) show statistically significant evidence of clustering, although the magnitude is small. The only previous investigation of spatial clustering of childhood leukaemia (CL) in a large dataset was conducted in the UK (Draper, 1990). This, too, reported statistically significant evidence of weak clustering. The UK analysis was subsequently extended to examine temporal patterns of cases within the areas that showed most evidence of clustering to test two specific prior hypotheses (Alexander, 1992), namely (a) that exposure to common infectious agents at certain critical times may cause CL; and (b) that the relevant exposures often occur in the context of a microepidemic in the geographical population; the origin and justification of these hypotheses are reviewed in Greaves and Alexander (1993) and Kinlen (1995). A second objective of EUROCLUS was to test these hypotheses and make comparisons with the results of the UK analysis (Alexander, 1992). Specific subgroups of interest are (a) cases of acute lymphoblastic leukaemia (ALL) in the childhood peak, and (b) cases of CL aged 5–14 years. Critical times, specified in advance are for (a) the 18-month period preceding diagnosis, and for (b), 1 year before to 1 year after birth.

A third subgroup of interest that had not been included in the UK analysis is infant leukaemia (diagnosed < 1 year or < 18 months), which has recently emerged as an interesting biological entity as a large proportion of these cases have rearrangement of

*Received 21 April 1997*
*Revised 27 July 1997*
*Accepted 10 September 1997*

*Correspondence to:* FE Alexander the *MLL* gene (Kumar, 1993). Because these rearrangements are rare in older subjects, except in cases of secondary leukaemia after epipodophyllotoxin therapy, it has been hypothesized that environmental exposures that, like the epipodophyllotoxins, inhibit the action of topoisomerase II may cause infant leukaemias (Ford et al, 1993; Ross et al, 1994). Twin studies have demonstrated that infant cases with *MLL* rearrangements arise in utero (Ford et al, 1993). Thus, the prevailing hypotheses for infant leukaemia, unlike those for older cases, do not relate to exposure to infectious agents. However, as the statistical significance of the EUROCLUS results depended on the inclusion of the infant cases (Alexander et al, 1998) the analyses were extended to include infants.

### METHODS

The method of analysis of spatial clustering applied to the EUROCLUS data was the Potthoff–Whittinghill method (Muirhead and Butland, 1996). The test statistic is based on the sum of statistics calculated for each small census area as $O(O-1)/E$, where $O$ and $E$ are the observed and expected numbers of cases. This does not lend itself to ranking of areas with highly variable values of $E$ as the largest values can occur in areas with large populations but with a deficit of observed compared with expected. Alternative methods of ranking based on $O/E$ and the Poisson $P$-values have been used conventionally, but encounter similar problems in that they are highly sensitive to variations in the expected numbers. A methodological study conducted for EUROCLUS (submitted) identified an alternative ranking statistic $[O(O-1)/E-E]$ that was superior in terms of sensitivity and specificity in identifying clustered areas. This has been used (see Appendix 1) to select up to 25 areas in each geographical region as being the most clustered. These areas form the basis of the present study. They have also been compared with 'control' areas; significant differences for population mixing and other demographic factors previously associated with CL (Kinlen, 1995) provide confirmation that the algorithm selects for study areas that are aetiologically meaningful (Alexander et al, submitted).

The present study population consists of all cases of CL diagnosed in the period 1980–89 in the areas defined as clustered in 14 countries that participated in this phase of EUROCLUS. These cases were divided into two diagnostic groups – ALL and other leukaemias. Cases of non-Hodgkin's lymphoma were ascertained for EUROCLUS, primarily for purposes of quality control; they were occasionally used in the selection of cluster areas (see Appendix 1), but have not been included in the present analysis. Three analyses have been conducted corresponding to different definitions of diagnostic group, age at diagnosis and period of risk of a critical group (series A1–A3) specified in advance.

### Series A1

Cases of ALL aged 2–4 years at diagnosis with risk period the 18-month period before diagnosis (or age 12 months to diagnosis if that is shorter). This group is defined in such a way as to represent the childhood peak of ALL, and also to ensure that the risk period was entirely distinct from the time around birth.

### Series A2

Cases of childhood leukaemia aged 5–14 years at diagnosis with risk period from 1 year before to 1 year after birth.

### Series A3

Cases of infant leukaemia (diagnosed ≤ 12 months) with risk period surrounding the time in utero: specifically 12 months before birth to 6 months after birth.

Linkages of children in series A1 with those in series B (all cases of CL in the clustered areas) were identified; these linkages can be of time and/or space. Two distinct cases, one taken from each series are linked in *time* if the risk period of the series A1 case overlaps the period from the date of conception (taken as date of birth –12 months) to date of diagnosis of the series B case by at least 3 months, and in *space* if the places of residence when diagnosed were in the same small area.

Similar analyses were carried out for series A2 and A3.

The overlap of 3 months is intended to reflect the fact that epidemics are not localized to just one moment in time. As linkage in time requires this overlap, the risk periods specified for each of Series A1–A3 extend the biologically most plausible periods of risk of exposure by around 3 months in each direction (for example the in utero risk period is taken to begin 12 months before birth, the prediagnosis risk period ends at diagnosis, although a few months must elapse between the causative exposure and diagnosis).

The analysis involves computation of the numbers of cases that are linked in both space and time after allowing for the numbers of cases linked separately in time or space (Knox, 1964). These linkages for pairs of cases for each country/region are shown diagrammatically below:

| | | Linked in time: Yes | Linked in time: No | |
|---|---|---|---|---|
| Linked in space | Yes | a | b | S |
| | No | c | d | N–S |
| | | T | N–T | N |

The expected number ($E$) of pairs linked in both space and time under the null hypothesis of no space–time interaction is ST/N. In the classical Knox test, there is just one series of cases and one time of risk and a is compared with $E$, using the approximating Poisson distribution or a permutation or Monte Carlo test in which S, T and N are all held fixed. The present analysis uses a modification of the Knox test identical to that of Alexander (1992). This uses a Monte Carlo procedure; the array of dates of birth/dates of diagnosis pairs of the entire study population were randomized within diagnosis group within country/region while keeping the small area of diagnosis arrays fixed. The numbers of cases in both diagnostic groups are thus held constant for individual small areas. Within each country/region, the following also remain constant: (a) the number of cases in each series, (b) the total number (N) of pairs of cases, (c) the number of these pairs (T) linked in time, (d) the age, time of birth and time of diagnosis distributions for each diagnostic group. However, the number of cases in series A1 (or A2, A3) in individual small areas will change and so the number of space linkages ($S$) and hence $E$ will vary [The randomization could have been applied separately to age groups within ALL, ANLL that would have kept $S$ fixed but the methodology of Alexander (1992) has been followed precisely. This also allows the use of identical randomization procedures in each analysis.] Monte-Carlo testing has been applied to the standardized $Z$ statistic of the Knox test that takes account of this:

© Cancer Research Campaign 1998

$$Z = \frac{(O-E)}{\sqrt{(E)}}$$

where $O$ is the observed number and $E$ the expected number of space–time linkages summed over countries/regions.

Although no assumptions are required for the *analysis* of the space–time data, it is prudent to apply two simple assumptions when *interpreting* the results in terms of exposure to an infection occurring at the place of residence (see also, Smith et al, 1976; Alexander, 1992).

*Assumption I*: The children have lived in the same small area throughout the time from birth to diagnosis (and their mothers have lived there during the relevant pregnancies).
*Assumption II*: A child who will subsequently be diagnosed with leukaemia is a marker of risk of exposure to a causative infection in the area in which he/she is currently living.

Under these assumptions, excess space–time interactions for analyses of series A1, A2 are testable predictions of prior hypotheses of infectious aetiologies for CL. Strictly, they are only required for children involved in space–time interactions at the (unknown) time of their aetiological exposures.

### RESULTS

The cases in the study population (Tables 1 and 2) are from 14 countries and show the usual high frequency of ALL at the childhood peak ages. Two countries (Switzerland and France) had fewer than 25 areas meeting the threshold for selection as clustered. The remainder all had 25 areas selected but the size (in terms of population at risk) of the areas varied by country and, in consequence, the number of cases is quite variable. The proportion of infant cases in the clustered areas is slightly higher than in the total dataset.

Results (Tables 3 and 4) show, for analyses of series A1 and A2, highly significant overall excesses of pairs linked in both space and time. The patterns are qualitatively present in the majority of individual countries, although certain ones are particularly influential: Spain, UK and Italy for series A1; and Australia and Spain for series A2. Results for Europe remain statistically significant. A small number of countries have a deficit of space–time pairs; these include Germany and Sweden for series A1 and Norway for series A2. Excess space–time pairs are seen for countries that did not show evidence of generalized clustering (Alexander et al, 1998) as well as those that did.

For series A3 (infant cases), the number of cases was much smaller and, in consequence, statistical power was reduced. The results (Table 5) show a larger and statistically significant percentage excess (26%) of observed to expected space–time pairs with individual excesses in each country apart from England and Wales based, often, on very small numbers.

Thus, the temporal distributions of the cases diagnosed in the clustered areas were non-random. Analyses for other definitions of series A, specifically, other sets of critical periods (in particular, young ALL cases around the time of birth, and older cases close to diagnosis) did not reveal evidence of space–time linkage.

### DISCUSSION

One clear conclusion follows from the present results: testing for the predicted space–time patterns has confirmed their presence and

**Table 1** Cases included, by age group and diagnosis

| | ALL | Other leukaemia | Total |
|---|---|---|---|
| Infants < 1 year | 33 | 21 | 54 |
| Others ≤ 4 years | 469[a] | 44 | 513 |
| 5–14 years | 347 | 56 | 403 |
| Total | 849 | 121 | 970 |

[a]One case in Sweden excluded as duplicate value of id, date of birth, area. This may be a duplicate case or a twin but in either event it is conservative to exclude from these analyses.

**Table 2** Cases included by country

| Country | Total number | ALL 2–4 years | Total leukaemia 5–14 years | Infants |
|---|---|---|---|---|
| Australia[a] | 77 | 37 | 33 | 3 |
| Denmark | 94 | 42 | 37 | 6 |
| England and Wales | 69 | 37 | 19 | 4 |
| Finland | 93 | 36 | 41 | 4 |
| France[b] | 18 | 6 | 9 | 0 |
| Germany | 77 | 29 | 35 | 5 |
| Greece | 94 | 44 | 36 | 4 |
| Italy[c] | 58 | 19 | 33 | 1 |
| Netherlands | 114 | 53 | 43 | 5 |
| Norway | 70 | 31 | 30 | 2 |
| Scotland | 72 | 31 | 27 | 7 |
| Spain[d] | 41 | 13 | 18 | 7 |
| Sweden | 84[e] | 27 | 36 | 6 |
| Switzerland[f] | 9 | 2 | 6 | 0 |

[a]Queensland; [b]Côte D'Or; [c]Piedmont; [d]Valencia. [e]One case in Sweden excluded since duplicate value of id, date of birth, area. This may be a duplicate case or a twin, but in either event it is conservative to exclude from these analyses. [f]Vaud and Neuchatel.

**Table 3** Space–time interactions: analysis of series A1[a]

| Country | Observed | Expected | Standardized z | Per cent excess |
|---|---|---|---|---|
| Australia | 45 | 41.99 | 0.47 | 7.2 |
| Denmark | 56 | 53.75 | 0.31 | 4.2 |
| England and Wales | 32 | 25.24 | 1.35 | 26.8 |
| Finland | 51 | 42.88 | 1.24 | 18.9 |
| France | 6 | 5.65 | 0.15 | 6.3 |
| Germany | 23 | 25.33 | –0.46 | –9.2 |
| Greece | 88 | 83.14 | 0.53 | 5.8 |
| Italy | 19 | 15.29 | 0.95 | 24.3 |
| Netherlands | 110 | 101.04 | 0.89 | 8.9 |
| Norway | 26 | 25.97 | 0.00 | 0.1 |
| Scotland | 29 | 22.52 | 1.37 | 28.8 |
| Spain | 9 | 6.38 | 1.04 | 41.2 |
| Sweden | 31 | 31.57 | –0.10 | –1.8 |
| Switzerland | 1 | 0.94 | 0.06 | 6.7 |
| Europe[b] | 481 | 434.69 | 2.22 | 10.7 |
| All countries[c] | 526 | 476.20 | 2.28 | 10.5 |

[a]ALL cases aged 2–4 years; time at risk, 18 months preceding diagnosis; [b]Monte Carlo *P*-values (based on 9999 runs), 0.0046; [c]Monte Carlo *P*-values (based on 9999 runs), 0.0050.

 © Cancer Research Campaign 1998

**Table 4** Space–time interactions: analysis series A2[a]

| Country | Observed | Expected | Standardized $z$ | Per cent excess |
|---|---|---|---|---|
| Australia | 49 | 30.50 | 3.35 | 60.6 |
| Denmark | 52 | 41.92 | 1.56 | 24.0 |
| England and Wales | 14 | 10.92 | 0.93 | 28.3 |
| Finland | 54 | 52.70 | 0.18 | 2.5 |
| France | 7 | 6.59 | 0.16 | 6.3 |
| Germany | 31 | 24.35 | 1.35 | 27.3 |
| Greece | 49 | 45.56 | 0.51 | 7.5 |
| Italy | 18 | 17.60 | 0.10 | 2.3 |
| Netherlands | 61 | 56.75 | 0.56 | 7.5 |
| Norway | 17 | 19.86 | –0.64 | –14.4 |
| Scotland | 19 | 16.37 | 0.65 | 16.1 |
| Spain | 9 | 5.63 | 1.42 | 59.9 |
| Sweden | 40 | 35.65 | 0.73 | 12.2 |
| Switzerland | 3 | 3.00 | 0.00 | 0.0 |
| Europe[b] | 374 | 337.66 | 1.98 | 10.8 |
| All Countries[c] | 423 | 368.75 | 2.82 | 14.7 |

[a]All leukaemias, age 5–14 years; time at risk, date of birth ± 1 year; [b]Monte Carlo $P$-values (based on 9999 runs), 0.0038; [c]Monte Carlo $P$-values (based on 9999 runs), 0.0002.

**Table 5** Space–time interactions: analysis series A3[a]

| Country | Observed | Expected | Standardized $z$ | Per cent excess |
|---|---|---|---|---|
| Australia | 3 | 2.26 | 0.49 | 32.6 |
| Denmark | 12 | 9.08 | 0.97 | 32.2 |
| England and Wales | 1 | 2.00 | –0.71 | -50.0 |
| Finland | 4 | 3.62 | 0.20 | 10.6 |
| France | | Not applicable[b] | | |
| Germany | 6 | 4.37 | 0.78 | 37.4 |
| Greece | 5 | 4.16 | 0.41 | 20.2 |
| Italy | 1 | 0.65 | 0.44 | 54.1 |
| Netherlands | 8 | 6.49 | 0.59 | 23.3 |
| Norway | 5 | 4.87 | 0.06 | 2.7 |
| Scotland | 6 | 4.39 | 0.77 | 36.5 |
| Spain | 2 | 1.69 | 0.24 | 18.6 |
| Sweden | 10 | 7.78 | 0.80 | 28.6 |
| Switzerland | | Not applicable[b] | | |
| Europe[c] | 60 | 47.47 | 1.82 | 26.4 |
| All countries[d] | 63 | 50.01 | 1.84 | 26.0 |

[a]Infant cases (diagnosed < 1 year old); time at risk, from 12 months before birth to 6 months after birth; [b]no cases in Series A3; [c]Monte Carlo $P$-values: (based on 9999 runs), 0.041; [d]Monte Carlo $P$-values (based on 9999 runs), 0.032.

the evidence is highly statistically significant. The results confirm those previously reported for ALL in UK data. The method, although based on that of Knox, addresses more specific aetiological hypotheses and is also more complex than tests of space–time clustering using time and place of diagnosis. The last tests have been applied widely to CL with somewhat equivocal conclusions (for example van Steensel-Moll et al, 1983) and positive results most frequent for young children (0–4 years) (Alexander, 1993). The Knox test has not been applied to the present data, with the exception of Greece where marked space–time interaction was observed for this youngest age group (Petridou et al, 1996).

As many animal leukaemias are caused by viruses (Onions, 1985) and some related human conditions by viruses and bacteria (Robert-Gurroff and Gallo, 1983; Wotherspoon et al, 1993; Schultz and Neil, 1996), the hypothesis that childhood leukaemia is caused by similar exposures is plausible. Studies by Kinlen and colleagues (reviewed in Kinlen, 1995) are difficult to explain, except in terms of increase in risk of leukaemia consequent upon exposure patterns that pertain when herd immunity is dysregulated by population mixing. These studies provide little guidance concerning critical times of exposure. Other epidemiological data provide persuasive, although indirect, evidence to support the hypothesis (Greaves and Alexander, 1993; Greaves, 1997) that delayed first exposure to an unknown infectious agent (or agents) may cause ALL at the childhood peak ages. The evidence includes the evolution of the peak as societies 'modernize', and increased risk for children likely to have been protected from early infection relative to their peers – with increases in firstborn children (Fraumeni and Miller, 1967), those substantially younger than their siblings (Kaye et al, 1991), those living in isolated areas (Alexander et al, 1990), and decreases in those hospitalized for infectious disease as infants (van Steensel-Moll et al, 1986) and those attending preschool groups as infants (Petridou et al, 1993). Series A1 was chosen to address the above hypothesis.

The second hypothesis of interest is that in utero or neonatal exposure to an infectious agent or agents contributes to CL in older children. The justification for this and the selection of series A2 in the original analysis (Alexander, 1992) were considerably weaker; they came directly from the analysis of residential histories in two case–control studies (Smith et al, 1976; Alexander et al, 1992) with indirect support coming from comparisons of the ages of the persons most likely to have been involved in mixing with the ages of the subsequent leukaemia excess in Kinlen's studies in which, in general, mixing of adults is associated with excess CL outside the childhood peak ages (reviewed in Alexander, 1993). Mixing of adults will most obviously influence children in utero or during early life.

To test these hypotheses using the present study design, some assumptions are required (see Methods). Assumption I is unlikely to be precisely true: some of the children will have moved between birth and diagnosis and, in particular, between contact with a causative infection and diagnosis. However, we have selected areas in which the excess leukaemia risk is likely to have a degree of permanence (indicated by the excess incidence calculated over a 10-year period) and in which, if the area has any aetiological significance, (most of) the affected children would have been living when a critical exposure occurred. The same selection of areas was applied for the same reason in the earlier analysis (Alexander, 1992).

We turn now to Assumption II. If infectious organisms can contribute to (some) cases of childhood leukaemia, then these cases will have been exposed at some time before their diagnosis. For some, perhaps most, of these children, exposure will have occurred in the area in which they were then living. It is reasonable to suppose that the opportunity for exposure of other children living in that area was increased at the same time. The child would be a marker of excess risk of exposure in the local community at this (unknown) point in time. As the time cannot be identified and latent periods may be highly variable, it is prudent to interpret this as marking excess risk 'on average' over the entire time from conception to diagnosis. This provides support for Assumption II, although social mobility will lead to exposures elsewhere. Selection of high-risk areas should, as has been argued above, minimize this. We note that the assumptions are strictly required only for a limited set of cases at specific times.

The present results, taken together with those of Alexander (1992) are, at least, consistent with the hypotheses outlined above

© Cancer Research Campaign 1998

and it is reasonable to argue that they offer support for them. If so, they suggest that the agents need not be age- nor subtype-specific, although critical times of exposure may well be specific. For the childhood peak of ALL, they point to *post-natal* rather than to *in utero* exposure, which has been proposed by Smith, 1997.

Other explanations for the present findings are certainly possible but rather contrived. These include ones involving environmental 'accidents' (i.e. short-term release of chemical or other pollutants). Exposures to cyclical epidemic infections could possibly be involved. Other analyses have indicated that a small number of the clustered areas are near to environmental hazards previously associated with increased incidence of CL (EUROCLUS, 1997).

Further clarification of these findings (in the absence of identification of causative agents) will require analyses of complete residential histories and these are in progress.

Analyses of infant cases (series A3) were not included in the previous study but the results are statistically significant and the percentage excess of space-time linkages both large and concentrated. Although exposure to infections is not often considered as a factor in the aetiology of infant leukaemia, one of Kinlen's studies found an excess only for children under the age of 2 years and the largest excess was for infants (Kinlen and Hudson, 1991); as noted above, EUROCLUS found infants to be intrinsically involved in spatial clustering, but the *MLL* status of the infant cases is unknown. Thus, the present results cannot be interpreted as indicative of an infectious aetiology for infant leukaemias having *MLL* gene rearrangements. They do nevertheless point to sharing of aetiological exposures by at least a minority of infant leukaemias with older cases and suggest that these exposures may be to common infectious agents. Characterization of the relevant infant subgroup must be an important research priority.

### ACKNOWLEDGEMENTS

The coordination of this project is funded by the European Union under its BIOMED programme of Concerted Actions as Project Number PL93-1785*26.02.1993. Dr Freda Alexander is also partially supported by the Leukaemia Research Fund and the Kay Kendall Leukaemia Fund. The Australian Paediatric Cancer Registry is supported by the Queensland Cancer Fund. The data for Greece were generated with contributions from Professor S Haida, Professor M Kalmanti, Dr D Koliouskas, Dr H Kosmidi and Dr F Piperopoulou. The Childhood Cancer Research Group is supported by the UK Departments of Health. The Childhood Cancer Registry of Piedmont is supported by the Italian National Council, contract 95.00449.PF39 and by the Italian Association for Cancer Research. The collaboration of Dr G Couillault and Dr Maynadie with Professor Carli is acknowledged. The Childhood Cancer Registry of Valencia is supported by the Conselleria de Sanitat i Consum of the Generalitat Valenciana. The contribution of Professor Boyle was within the framework of support of the Associazione Italiana per la Ricerca sul Cancro (AIRC). Dr Colin Muirhead is thanked for his contributions to meetings of the Statistics Subcommittee for this project. Mrs Rosemarie Bland and Miss Patricia Bisset are thanked for preparing the manuscript and Mr Darren Downing for assisting with computer graphics.

### REFERENCES

Alexander FE (1992) Space-time clustering of childhood acute lymphoblastic leukaemia: indirect evidence for a transmissible agent. *Br J Cancer* **65**: 589–592

Alexander FE (1993) Viruses, clusters and clustering of childhood leukaemia: a new perspective? *Eur J Cancer* **29**: 1424–1443

Alexander FE, Ricketts TJ, McKinney PA and Cartwright RA (1990) Community lifestyle characteristics and risk of acute lymphoblastic leukaemia in children. *Lancet* **336**: 1461–1464

Alexander FE, McKinney PA, Moncrieff K and Cartwright RA (1992) Residential proximity of children with leukaemia and non-Hodgkin's lymphoma in three areas of Northern England. *Br J Cancer* **65**: 583–588

Alexander FE, Wray N, Boyle P, Carli P-M, Coebergh JW, Draper G, Ekbom A, Levi F, McKinney PA, Michaelis J, Petridou E, Peris-Bonet R, Pukkala E, Storm H, Terracini B and Vatten L on behalf of the EUROCLUS project (1996) Clustering of childhood leukaemia: a European study in progress. *J Epidemiol Biostatist*, **1**: 13–24.

Alexander FE, Boyle P, Carli P-M, Coebergh JW, Draper GJ, Ekbom A, Levi F, McKinney PA, McWhirter W, Michaelis J, Peris-Bonet R, Petridou E, Pompe-KirnV, Plesko I, Pukkala E, Rahu M, Storm H, Terracini B, Vatten L and Wray N, on behalf of the EUROCLUS project. (1998). Spatial clustering of childhood leukaemia: summary results from the EUROCLUS project. *Br J Cancer*

Alexander FE, Boyle P, Carli P-M et al. Demographic factors in small areas containing clusters of childhood leukaemia: results of the EUROCLUS study. (submitted).

Doll R (1989) The epidemiology of childhood leukaemia. *J Royal Statist Soc Series A* **152**: 341–351

Draper GJ (1990) *The Geographical Epidemiology of Childhood Leukaemia and non-Hodgkin Lymphomas in Great Britain, 1966–83*. OPCS, HMSO: London

EUROCLUS 1997 Clustering of childhood leukaemia in Europe – final report. Department Public Health Services: University of Edinburgh

Ford AM, Ridge SA, Cabrera ME, Mahmoud H, Steel CM, Chan LC and Greaves MF (1993) In utero rearrangements in the trithorax-related oncogene in infant leukaemias. *Nature* **363**: 358–360

Fraumeni JF and Miller RW (1967) Epidemiology of human leukaemia: recent observations. *J Natl Cancer Inst* **38**: 593–605

Greaves MF (1997) Aetiology of acute leukaemia. *Lancet* **349**: 344–349

Greaves MF and Alexander FE (1993) An infectious etiology for common acute lymphoblastic leukemia in childhood? *Leukaemia* **7**: 349–360

Kaye SA, Robison LL, Smithson WA, Gunderson P, King FL and Neglia JP (1991) Maternal reproductive history and birth characteristics in childhood acute lymphoblastic leukaemia. *Cancer* **68**: 1351–1355

Kinlen LJ (1995) Epidemiological evidence for an infective basis in childhood leukaemia. *Br J Cancer* **71**: 1–5

Kinlen LJ and Hudson C (1991) Childhood leukaemia and poliomyelitis in relation to military encampments in England and Wales in the period of national military service. *Br Med J* **303**: 1357–1362

Knox EG (1964) The detection of space-time interactions. *Appl Statist* **13**: 25–29

Kumar L (1993) Epipodophyllotoxin-related secondary leukaemia. *Lancet* **342**: 819–820

Muirhead CR and Butland BK (1996) Testing for over-dispersion using an adapted form of the Potthoff-Whitinghill method. In *Methods for Investigating Localized Clustering of Disease*. Alexander FE and Boyle P (eds) pp. 40–54 IARC Scientific Publication No. 135: Lyon

Onions D (1985) Animal models: lessons from feline bovine leukaemia virus infections. *Leukaemia Research* **9**: 709–11

Parkin DM, Stiller CA and Draper GJ (1988). *International Incidence of Childhood Cancer*. Publication No 87: Lyon

Petridou E, Kassimos D, Kalmanti M, Kosmidis H, Hardas S, Flytzani V, Tong D and Trichopoulous D (1993) Age of exposure to infections and risk of childhood leukaemia. *Br Med J* **307**: 774

Petridou E, Revinthi K, Alexander FE, Haidas S, Koliouskas D, Kosmidis H, Piperopoulou F, Tzortzatou F and Trichopoulous D (1996) Space–time clustering of childhood leukaemia in Greece: evidence supporting a viral etiology. *Br J Cancer* **73**: 1278–1283

Robert-Guroff M and Gallo RC (1983) Establishment of an etiologic relationship between the human T-cell leukemia/lymphoma virus (HTLV) and adult T-cell leukemia. *Blood* **47**: 1–12

Ross JA Potter JD, Robinson LL (1994) Infant leukaemia, topoisomerase II inhibitors, and the MLL gene. *J Natl Canc Inst* **86**: 1678–1680

Schluz TF and Neil JC (1996) Viruses and Leukaemia. In: *Leukaemia* 6th edn, Henderson ES, Listen TA and Greaves MF (eds), pp. 160–178 W B Saunders: Philadelphia

Smith PG, Pike MC, Till MM and Hardisty RM (1976) Epidemiology of childhood leukaemia in Greater London: a search for evidence of transmission assuming a possibly long latent period. *Br J Cancer* **33**: 1–8

Smith M (1997) Considerations on a possible viral etiology for B-precursor acute lymphoblastic leukaemia of childhood. *J Immunotherapy* **20**: 89–100

© Cancer Research Campaign 1998

Van Steensel-Moll HA, Valkenburgh HA, Vandenbroucke JP and Van Zenen GE (1983) Time-space distribution of childhood leukaemia in the Netherlands. *J Epidemiol Comm Health* **37:** 145–148

Van Steensel-Moll HA, Valkenburg HA and Van Zanen GE (1986) Childhood leukaemia and infectious diseases in the first year of life: a register based case-control study. *Am J Epidemiol* **124:** 590–594

Wotherspoon AC, Doglioni C, Diss TC, Pan LX, Moschini A, Deboni M and Issacson PG (1993) Regression of primary low-grade B-cell gastric lymphoma of mucosa-associated lymphoid tissue type after eradication of *Helicobacter pylori. Lancet* **342:** 575–577

### APPENDIX 1: SELECTION OF CLUSTERED AREAS

Selection was performed on an individual country (or region) basis and was limited to areas with between 0.1 and 5.0 expected cases of total childhood leukaemia in the period 1980–89. This criterion was chosen to exclude both extremely small and extremely large areas.

For each diagnosis group [acute lymphoblastic lymphoid leukaemia (ALL) and total childhood leukaemia (CL) at ages 1–7 years, 0–4 years, 0–14 years and lymphoid leukaemia/non-Hodgkin's lymphoma (LL/NHL) at ages 0–14 years] within each area the value of the ranking statistic

$$W = \frac{O(O-1)}{E-E}$$

was calculated and allocated to a value category (> 28, 20–28, 14–20, 10–14, 8–10, 6–8, 4–6, 2–4, 0–2). The use of $W$ as ranking statistic for the selection of clustered areas was justified by a methodological study conducted for EUROCLUS (Wray et al, submitted). Values of $W < 0$ were not included in the ranking. The maximum value of $W$ differed between countries – from 15 (Denmark) to 106 (England and Wales). Area diagnosis groups that were in the same $W$-category were ordered by the $P$-value of the Potthoff–Whittinghill test for the country/region and diagnostic group (categories being < 1%, 1–5%, 5–10%, > 10%). Finally, any area diagnosis groups that were in the same $W$-category and $P$-value category were ordered by age-diagnostic group priority (based on prior hypotheses). These were ALL 1–7 years, ALL 0–4 years, CL 1–7 years, CL 0–4 years, ALL 0–14 years, TL 0–14 years, ALL/NHL 0–14 years. This provided an unambiguous ranking of selected small areas – diagnosis groups pairs in each country/region with no ties. The first 25 areas occurring in this list were selected as clustered areas for the country/region (some areas were ranked highly for several diagnosis groups). Smaller numbers of areas were selected when a country/region had < 25 areas with $W > 0$ (Cote d'Or, France and Vaud and Neuchatel, Switzerland).

### APPENDIX 2: COLLABORATORS IN THE EUROCLUS PROJECT

| | |
|---|---|
| Australia | Cancer Registry of Queensland<br>Dr W McWhirter |
| Denmark | Danish Cancer Registry<br>Dr H Storm<br>Dr JH Olsen |
| England and Wales | Childhood Cancer Group<br>Dr GJ Draper<br>Dr CA Stiller |
| Estonia | Department of Epidemiology and Biostatistics<br>Institute of Experimental and Clinical Medicine<br>Professor M Rahu |
| Finland | Finnish Cancer Registry<br>Dr E Pukkala<br>Dr L Teppo |
| France | Registry of Haematopoietic Malignancies<br>Professor PM Carli<br>Dr G Couillault<br>Dr M Maynadié |
| Germany | National Register of Childhood Malignancies<br>Professor Dr J Michaelis<br>Dr I Schmidtmann |
| Greece | Special Data Collection<br>Dr E Petridou |
| Italy | European Institute of Oncology<br>Professor P Boyle<br>Childhood Cancer Registry of Piedmont<br>Professor B Terracini<br>Dr C Magnani |
| Netherlands | Dutch Childhood Leukaemia Study Group<br>Dr A Van Der-Does-Van Den Berg<br>Department of Epidemiology and Biostatistics,<br>Erasmus University<br>Dr JW Coebergh |
| Norway | Norwegian Cancer Registry<br>Dr L Vatten |
| Scotland | Co-ordinating Centre<br>Dr F E Alexander<br>Dr N Wray<br>Scottish Cancer Registry<br>Dr D Brewster<br>Dr P McKinney |
| Slovakia | The National Cancer Registry of Slovakia<br>Dr I Plěsko |
| Slovenia | Cancer Registry of Slovenia<br>Professor Dr V Pompe-Kirn |
| Spain | Childhood Tumour Registry of Valencia<br>Dr R Peris-Bonet |
| Sweden | Department of Cancer Epidemiology, University of Uppsala<br>Dr H-O Adami<br>Dr A Ekbom<br>Swedish Cancer Registry<br>Dr J Bring |
| Switzerland | Registres Vaudois et Neuchatelois des Tumeurs<br>Dr F Levi |

© Cancer Research Campaign 1998